# IVIG Response Is Not Diagnostic: Strengthening Dengue‐Kawasaki Overlap Reports With Objective Treatment Triggers and Serial Echocardiography

**DOI:** 10.1002/ccr3.73096

**Published:** 2026-07-03

**Authors:** Muhammad Abdullah Awan, Hammad‐ud‐din Raza

**Affiliations:** ^1^ Wah Medical College, National University of Medical Sciences Rawalpindi Pakistan; ^2^ Wah Medical College Rawalpindi Pakistan

Dear Editor,

We read with great interest the case report by Toufeeq et al., titled “Secondary Dengue Infection Presenting With Kawasaki Disease‐Like Features: A Diagnostic Dilemma in a 12‐Year‐Old Girl,” published in Clinical Case Reports [1]. The authors present a clinically important case from a dengue‐endemic setting in which a 12‐year‐old girl developed prolonged fever, rash, conjunctivitis, strawberry tongue, edema, lymphadenopathy, thrombocytopenia, marked transaminitis, and positive dengue IgM and IgG serology. The report is valuable because it highlights the difficult overlap between classical Kawasaki disease (KD), dengue‐associated KD‐like inflammation, and possible concomitant disease.

However, one point deserves further emphasis: response to intravenous immunoglobulin (IVIG) should not be treated as diagnostic confirmation of KD. The patient became afebrile within 14 h of IVIG administration, with subsequent improvement in rash, conjunctivitis, edema, platelet trend, and liver enzymes [1]. While this clinical response supports the reasonableness of urgent treatment when KD cannot be excluded, it does not prove that the underlying diagnosis was classical KD. IVIG has broad immunomodulatory effects, and dengue itself has a dynamic febrile‐to‐defervescence course; therefore, improvement after IVIG may reflect treatment effect, natural disease evolution, or both.

The case therefore raises an important reporting issue for dengue‐endemic regions: clinicians need objective triggers for when IVIG should be administered despite confirmed or suspected dengue. In the reported patient, IVIG was given because the child fulfilled clinical KD criteria and initial echocardiography was normal but not sufficient to exclude evolving coronary disease [1]. This is clinically defensible. Nevertheless, future reports would be strengthened by explicitly listing the treatment decision thresholds: illness day, persistence of fever beyond 5 days, number of KD criteria fulfilled, CRP/ESR trajectory, platelet trend, degree of thrombocytopenia, liver enzyme elevation, bleeding risk, dengue warning signs, and echocardiographic findings.

A second important issue is aspirin and antiplatelet decision‐making. Toufeeq et al. appropriately deferred high‐dose aspirin because of severe thrombocytopenia and bleeding risk in the setting of dengue infection [1]. This is a useful clinical point, but it would benefit from being framed as a structured risk–benefit decision rather than a simple deviation from KD protocols. In dengue‐KD overlap, the need to prevent coronary complications must be balanced against hemorrhagic risk, hepatic injury, and platelet nadir. Reporting these variables in a decision table may help readers understand why IVIG was prioritized while aspirin was withheld.

Serial echocardiography is also central to the interpretation of this case. The authors report a normal acute echocardiogram and a normal 6‐week follow‐up echocardiogram [1]. This follow‐up is reassuring and strengthens the outcome assessment. However, because coronary artery abnormalities can evolve after the acute febrile phase, future dengue‐KD mimic reports should predefine echocardiographic follow‐up intervals and report *z*‐scores where available. A normal initial study should reduce immediate concern but should not prematurely close the diagnostic question.

The educational value of the case is strongest when framed as a diagnostic dilemma rather than as proof that secondary dengue and KD were definitively coexisting. The patient's age, severe thrombocytopenia, marked transaminitis, hepatosplenomegaly, and dengue serology strongly support dengue as a major driver of the presentation [1]. In contrast, mucocutaneous findings and prolonged fever justified concern for KD. A cautious formulation—dengue infection with KD‐like inflammatory features requiring empiric IVIG because KD could not be safely excluded—may therefore be more clinically precise.

Overall, Toufeeq et al. contribute an important case that is highly relevant to clinicians practicing in dengue‐endemic regions [1]. Its main lesson should be that IVIG may be justified in selected dengue‐associated KD‐like presentations, but response to IVIG alone should not be used as diagnostic proof. Standardized treatment triggers, aspirin safety criteria, and serial echocardiographic follow‐up would make future reports more reproducible and clinically actionable.

## 1 Conclusion

This case highlights the diagnostic uncertainty created when secondary dengue infection presents with Kawasaki‐like features. IVIG may be appropriate when KD cannot be excluded, but clinical response after IVIG is not diagnostic. Future reports should define objective IVIG triggers, document aspirin risk–benefit reasoning, and include planned serial echocardiography to improve decision‐making in dengue‐endemic settings.

## Author Contributions

**Muhammad Abdullah Awan:** conceptualization, data curation, formal analysis, project administration, writing – original draft, writing – review and editing, supervision. **Hammad‐ud‐din Raza:** writing – original draft, writing – review and editing, formal analysis.

## Funding

The authors have nothing to report.

## Ethics Statement

The authors have nothing to report.

## Consent

The authors have nothing to report.

## Conflicts of Interest

The authors declare no conflicts of interest.

## Data Availability

No new data were generated or analyzed for this letter.
